# Estimation of *Trans* Fatty Acid Intake in Japanese Adults Using 16-Day Diet Records Based on a Food Composition Database Developed for the Japanese Population

**DOI:** 10.2188/jea.JE20090080

**Published:** 2010-03-05

**Authors:** Mai Yamada, Satoshi Sasaki, Kentaro Murakami, Yoshiko Takahashi, Hitomi Okubo, Naoko Hirota, Akiko Notsu, Hidemi Todoriki, Ayako Miura, Mitsuru Fukui, Chigusa Date

**Affiliations:** 1 Department of International Health, Graduate School of Medicine, The University of Tokyo, Tokyo, Japan; 2 Department of Social and Preventive Epidemiology, School of Public Health, The University of Tokyo, Tokyo, Japan; 3 Department of Health and Nutrition, School of Home Economics. Wayo Women’s University, Ichikawa, Chiba, Japan; 4 Department of Social and Preventive Epidemiology, Graduate School of Medicine, The University of Tokyo, Tokyo, Japan; 5 Department of Health and Nutritional Science, Faculty of Human Health Science, Matsumoto University, Matsumoto, Nagano, Japan; 6 Department of Food Science and Nutrition, Tottori College, Kurayoshi, Tottori, Japan; 7 Department of Public Health and Hygiene, School of Medicine, University of the Ryukyus, Naha, Japan; 8 Institutional affiliation: Department of Health and Nutritional Science, Faculty of Health Promotional Science, Hamamatsu University, Hamamatsu, Shizuoka, Japan; 9 Laboratory of Statistics, School of Medicine, Osaka City University, Osaka, Japan; 10 Department of Food Science and Nutrition, Faculty of Human Life and Environment, Nara Women’s University, Nara, Japan

**Keywords:** food composition database, *trans* fatty acids, Japanese population

## Abstract

**Background:**

The Standard Tables of Food Composition in Japan do not include information on *trans* fatty acids. Previous studies estimating *trans* fatty acid intake among Japanese have limitations regarding the databases utilized and diet assessment methodologies. We developed a comprehensive database of *trans* fatty acid food composition, and used this database to estimate intake among a Japanese population.

**Methods:**

The database was developed using analytic values from the literature and nutrient analysis software encompassing foods in the US, as well as values estimated from recipes or nutrient compositions. We collected 16-day diet records from 225 adults aged 30 to 69 years living in 4 areas of Japan. *Trans* fatty acid intake was estimated based on the database and the 16-day diet records.

**Results:**

Mean total fat and *trans* fatty acid intake was 56.9 g/day (27.7% total energy) and 1.7 g/day (0.8% total energy), respectively, for women and 66.8 g/day (25.5% total energy) and 1.7 g/day (0.7% total energy) for men. *Trans* fatty acid intake accounted for greater than 1% of total energy intake, which is the maximum recommended according to the World Health Organization, in 24.4% of women and 5.7% of men, and was particularly high among women living in urban areas and those aged 30–49 years. The largest contributors to *trans* fatty acid intake were confectionaries in women and fats and oils in men.

**Conclusions:**

Although mean *trans* fatty acid intake was below the maximum recommended intake of the World Health Organization, intake among subgroups was of concern. Further public health efforts to reduce *trans* fatty acid intake should be encouraged.

## INTRODUCTION

Industrially produced *trans* fatty acids, formed during the partial hydrogenation of commercial liquid vegetable oils to semi-solid fats, are found in margarine, shortening, and frying fats. Intake of these *trans* fatty acids is associated with metabolic and inflammatory risk factors and diseases, including coronary heart disease.^1^^–^^3^ Although small amounts are also found in ruminants as a result of biohydrogenation of polyunsaturated fatty acids, the few studies investigating such naturally derived *trans* fatty acid intake have found no association with some risk factors or with coronary heart disease,^2^^–^^5^ although the results were inconsistent.^4^ The World Health Organization (WHO) recommends that *trans* fatty acid intake be limited to less than 1% of total energy intake.^6^ Several Western countries have taken action to regulate consumption^7^: Denmark became the first country to ban fats and oils with greater than 2% industrially produced *trans* fatty acids in 2003^8^; the Netherlands has set an upper intake level of *trans* fatty acids of 1% of total energy intake^9^; and the Unites States (US) has mandated *trans* fatty acid listing on food labels^10^ and recommended that intake be as low as possible.^11^

Any investigation of the effects of *trans* fatty acid in specific populations should begin with the estimation of *trans* fatty acid intake in that population. However, few data are available on individual mean intakes estimated using *trans* fatty acid food composition tables covering foods high in *trans* fatty acids^1^^,^^12^^,^^13^ in Asian countries, including Japan. Several estimates for Japanese populations have been reported,^14^^–^^19^ but their usefulness is limited by problems with databases,^14^^–^^18^ dietary assessment methodologies,^15^^–^^18^ or sample sizes.^15^^,^^19^ Thus, our aim in this study was to develop a comprehensive *trans* fatty acid database that encompasses broader categories of foods, and then to estimate *trans* fatty acid intake among a Japanese population by using 16-day diet records (DR).

## METHODS

### Development of a *trans* fatty acid database

#### Number of food items and data sources

We developed a *trans* fatty acid database for 1995 foods: 1976 foods appearing in the Standard Tables of Food Composition in Japan (STFCJ)^20^ and 19 foods added in the present study. Among these 1995 foods, 1469 were found to contain no *trans* fatty acids (because they contained no or only a trace amount of fat^20^) and no industrially produced hydrogenated oils or ruminants.^12^^,^^13^^,^^21^^–^^33^

The primary data source for *trans* fatty acid values for the remaining 526 foods was direct chemical analysis.^17^^–^^19^^,^^21^^–^^33^ For this, we searched the Pubmed, CiNii, and Medical Online Library databases for the English and Japanese literature reporting analyses conducted in Japan of the *trans* fatty acid content of foods. For the present study, we limited data to reports appearing after the year 1992 (ie, data reported during the decade preceding collection of the present diet records) in order to minimize the possibility of changes in the nutrient composition of food products. Using these articles and their reference lists, we selected articles that assessed *trans* fatty acid content in foods by gas chromatography only.^34^ One article that did not indicate the number of samples analyzed^32^ was included, but was excluded from calculation when identical foods were analyzed in multiple articles, since the calculation of a mean value required the number of samples to be known. Further, we included data on analytic *trans* fatty acid values of 3 foods provided in the STFCJ^20^ (soft margarine, fat spread, and shortening) and unpublished data on 2 foods referenced in the STFCJ (Maruyama T, personal communication).

In addition, we reviewed the literature of other countries reporting foods with a high *trans* fatty acid content^12^^,^^13^^,^^35^^–^^39^ and selected those foods that were not included in the STFCJ.^20^ This process identified 19 foods determined to be important sources of *trans* fatty acids for addition to the database, including fast foods (*n* = 11), baked goods (*n* = 5), and confectionaries (*n* = 3). Fast foods were added because the STFCJ^20^ included only 1 fast food item (french fries) and omitted others (eg, hamburgers and fried chicken) produced by the major fast food chains.^40^ Regarding baked goods and confections, although 15 baked goods and 150 confectionaries were included in the STFCJ, we did not include other top-selling baked goods (eg, pastry with icing and muffins) and confectionaries (eg, almond chocolate and chocolate cake)^41^ with high levels of *trans* fatty acids.

When analytic data were unavailable, as a secondary data source we used data from the ESHA Food Processor SQL, which covers more than 35 000 foods, including food products and fast foods sold in the US (ESHA Research, Salem, Oregon),^42^ followed by a recipe book,^43^ or nutrient composition in the STFCJ.^20^

### Determination of the *trans* fatty acid content of 526 foods

Determination was done in a 4-step process, as follows:

**Step 1:** Assigning analytic values reported in the literature

*Trans* fatty acid values in the analytic data^17^^–^^19^^,^^21^^–^^33^ were converted to grams per 100 g of food, adjusting for total fat content in the STFCJ^20^ using the following equation: *Trans* fatty acid (g)/100 g of food = [*trans* fatty acid (g)/total fat (g) in reference] × [total fat (g)/100 g of food in the STFCJ]. For any reference that reported the *trans* fatty acid value for a specific food as % fat without indicating the fat content (g/100 g) of the food, we used the fat content (g/100 g) provided in the STFCJ^20^ to calculate the *trans* fatty acid value of the food.

We then considered a strategy to determine the *trans* fatty acid content of individual foods. Several articles analyzed the same type of food using the same method but provided different mean values. Discrepancies arose from variations in *trans* fatty acid content among food products. Also, most articles provided mean, minimum, and maximum values for analyzed products. In these cases, *trans* fatty acid content in individual foods might have been determined by choosing the highest or lowest mean value of multiple reports or by choosing the minimum or maximum value of the reports. To deal with these complexities, the following guidelines were applied.

1) When only 1 article existed and this article analyzed the *trans* fatty acid content in a single example of a food only, this value was selected (*n* = 13).

2) When only 1 article existed and this article analyzed the *trans* fatty acid content of several samples and reported minimum, maximum, and/or mean values, we selected the mean value for the food (*n* = 71).

3) When multiple articles existed but reported different mean *trans* fatty acid values for a specific food, we calculated the mean value by weighting the number of foods analyzed in each article (*n* = 59).

**Step 2:** Assigning analytic values to similar foods

2-1A: When the *trans* fatty acid value for a specific food (except meat cuts) could not be obtained using Step 1, but an analytic value had been obtained (using Step 1) for a similar food within the same food category of the same food group, that value (*trans* fatty acid % of total fat) was assigned after comparison with nutrient content (total energy and macronutrients) in the STFCJ^20^ (*n* = 102).

2-1B: When the *trans* fatty acid value for a specific food (except meat cuts) could not be obtained using Step 1, but an analytic value was available for a similar food within the same food group by Step 1, that value (*trans* fatty acid % of total fat) was assigned (*n* = 78).

2-2A: When the analytic *trans* fatty acid value of a specific meat cut was unavailable (Step 1), but an analytic value for the same part of the animal but with a different nutrient composition was, that value was assigned (*n* = 22).

2-2B: When the analytic *trans* fatty acid value of a specific meat cut was unavailable (Step 1), but an analytic value of a similar part of the animal having a similar nutrient composition was, that value was assigned (*n* = 17).

2-2C: When the analytic *trans* fatty acid value of a specific type of animal was unavailable (Step 1), but an analytic value of a similar type of animal having a similar nutrient composition and belonging to the same species was, that value was assigned (*n* = 37).

2-2D: When the analytic *trans* fatty acid value of a specific type of animal was unavailable (Step 1), but an analytic value of a different type of animal belonging to a different species was, that value was assigned (*n* = 7).

2-2E: When the analytic *trans* fatty acid value of a specific meat cut was unavailable (Step 1), but an analytic value of the same meat group was, that value was assigned (*n* = 88).

**Step 3:** Assigning values obtained from the ESHA Food Processor

For food products whose *trans* fatty acid values were unavailable using Steps 1 and 2, but for which a manufacturer was present in both Japan and the US, we compared the nutrient composition of the food in Japan, as shown on the website of the company, with that of the US, as provided in the ESHA Food Processor SQL, which covers more than 35 000 foods, including food products and fast foods sold in the US. The analytic nutrient values in ESHA databases were compiled from the latest US Department of Agriculture Standard Reference database, selected items from the Continuing Survey of Food Intakes by Individuals survey database, manufacturer data, data from fast food companies, and data from literature sources.^42^ For foods whose nutrient compositions (total energy and macronutrients) were similar (eg, fast foods, cookies, and a cornflake product), we assigned the value obtained from the ESHA (*n* = 14).

**Step 4:** Assigning values estimated from recipes and nutrient compositions

When the *trans* fatty acid values for a specific food were unavailable using Steps 1–3, we then imputed values by referring to recipes^43^ and the nutrient composition (total energy and macronutrients) of foods^20^ (*n* = 16). Among 16 foods, 4 foods were found to contain *trans* fatty acids (roast beef, beef jerky, and 2 types of Japanese omelet).

A summary of the number of foods determined at each step is shown in Table 1.

**Table 1.** Number of food items found to contain trans fatty acids

Food group^d^	Database development step^a,b,c^										

	1	2							3	4	Total

		1A	1B	2A	2B	2C	2D	2E			
Confectionaries (I)	25 (2)	49 (8)	34 (0)	0	0	0	0	0	2 (1)	12 (12)	122 (23)
Bakery (I)	6 (0)	12 (0)	9 (0)	0	0	0	0	0	1 (1)	0	28 (1)
Fats and oils (N)	11 (0)	2 (0)	6 (0)	0	0	0	0	0	0	0	19 (0)
Fats and oils (I)	5 (0)	0	0	0	0	0	0	0	0	0	5 (0)
Instant and retort foods (I)	17 (0)	26 (2)	0	0	0	0	0	0	0	0	43 (2)
Milk and dairy products (N)	23 (0)	13 (0)	8 (0)	0	0	0	0	0	0	0	44 (0)
Milk and dairy products (I)	7 (0)	0	0	0	0	0	0	0	0	0	7 (0)
Meat and meat products (N)	41 (0)	0	21 (0)	22 (0)	17 (0)	38 (0)	7 (0)	88 (0)	0	2 (0)	235 (0)
Margarine (I)	3 (0)	0	0	0	0	0	0	0	0	0	3 (0)
Fast foods (I)	1 (0)	0	0	0	0	0	0	0	10 (0)	0	11 (0)
Miscellaneous (I)	4 (0)	1 (0)	0	0	0	0	0	0	1 (0)	2 (0)	8 (0)
Total	143 (2)	103 (10)	78 (0)	22 (0)	17 (0)	38 (0)	7 (0)	88 (0)	14 (2)	16 (12)	526 (26)

(I) = industrially derived; (N) = naturally derived.

^a^Among a total of 1995 food items (ie, 1976 food items appearing in the Standard Tables of Food Composition in Japan^20^ and 19 brand food items), 1469 foods [ie, others including confectionaries (*n* = 14); instant and retort foods (*n* = 4); milk and dairy products (*n* = 1); meat and meat products (*n* = 4); vegetables (*n* = 472); fruits (*n* = 120); sugar (*n* = 33); fish (*n* = 416); rice and grains (*n* = 88); noodles (*n* = 32); nuts and pulses (*n* = 108); seasonings (*n* = 66); eggs (*n* = 18); beverages (*n* = 92); miscellaneous (*n* = 1)] were determined to contain no trans fatty acids as they contained no or only trace amounts of fat, no partially hydrogenated oils, and no ruminant products.

^b^Step 1: foods determined by analytic values; Step 2: foods determined by assigning the value of a similar food obtained from Step 1 [ie, 1A (foods other than meat cuts): value of a similar food within the same food category of the same food group was assigned; 1B (foods other than meat cuts): value of a similar food within the same food group was assigned; 2A (meat cuts): value of the same cut but different nutrient composition was assigned; 2B (meat cuts): value of a similar cut having a similar nutrient composition was assigned; 2C (meat cuts): value of a similar type of animal having similar nutrient composition and belonging to the same species was assigned; 2D (meat cuts): value of a different type of animal belonging to a different species was assigned; 2E (meat cuts): value of a same meat group was assigned]; Step 3: foods determined using the ESHA Food Processor SQL; and Step 4: foods determined by recipe or nutrient composition.

^c^Numbers in parentheses are the number of food items determined to have zero trans fatty acids in Steps 1–4.

^d^Confectionaries (I) include cookies, biscuits, yeast doughnuts, pies, tarts, cakes, traditional Japanese sweets, potato chips, crackers, other Japanese snacks, and chocolate; bakery (I) includes bread (eg, white, whole, rye, and French), danish, pastry, and cake doughnuts; fats and oils (N) include butter, lard, and beef tallow; fats and oils (I) include mayonnaise, salad dressing, and vegetable oils; instant and retort foods (I) include instant cooking sauce (eg, curry roux and stew roux), retort foods (eg, retort curry, retort stew, retort Chinese sauce), frozen foods, instant soup (eg, powder soup and cube bouillon), and instant noodles (eg, cup noodles); milk and dairy products (N) include milk, cheese, yogurt, ice cream, and lactic acid drinks; milk and dairy products (I) include partially hydrogenated coffee creamer, partially hydrogenated powder coffee creamer, partially hydrogenated cream, and partially hydrogenated whip cream; meat and meat products (N) include beef and poultry (eg, chicken, pork, sausages, ham, and organ meats); margarine (I) includes soft-type margarine, shortening, and fat spread; fast foods (I) include french fries, hamburgers, chicken burgers, fish burgers, and fried chicken; and miscellaneous (I) includes a fish product (fish paste), a grain product (corn flake), tofu products (eg, fried tofu); and egg products (eg, omelets).

### Estimation of *trans* fatty acid intake among a Japanese population

#### Study population

The study was conducted between November 2002 and September 2003 in 4 areas in Japan: Osaka (Osaka City), Okinawa (Ginowan City), Nagano (Matsumoto City), and Tottori (Kurayoshi City). In each area, we first recruited apparently healthy women aged 30 to 69 years who were living together with their husbands and willing to participate with their husbands without consideration to their husband’s age. Our recruitment strategy was to obtain 8 women for each 10-year age stratum (30–39 years, 40–49 years, 50–59 years, and 60–69 years). Before the study, the subjects attended group orientations, during which the study purpose and protocol were explained. Written informed consent was obtained from each subject. Body height was measured to the nearest 0.1 cm with the subject standing without shoes. Body weight in light indoor clothing was measured to the nearest 0.1 kg. Body mass index was calculated as body weight in kilograms divided by the square of body height in meters. A total of 121 women and 121 men completed the study protocol. For the analyses, we excluded women whose body weight was obviously mistyped in the database and men younger than 30 or older than 69 years (*n* = 11). Further, we excluded outliers of *trans* fatty acid intake, ie, those below or above the mean ± 3 standard deviations (g/day or % total energy), which left 119 women and 106 men aged 30 to 69 years for analysis.

#### Diet records

Subjects completed a 4-nonconsecutive-day semi-weighed DR for each season, at intervals of approximately 3 months: DR1 in November or December 2002 (autumn), DR2 in February 2003 (winter), DR3 in May 2003 (spring), and DR4 in August and September 2003 (summer). Each set of 4 recording days consisted of 1 weekend day and 3 weekdays. Details of the diet record procedure are provided elsewhere.^44^ Briefly, during the orientation session, registered dietitians gave the subjects both written and verbal instructions on how to keep the DR, provided recording sheets and a digital scale, and asked them to record and weigh all foods and beverages consumed on each recording day. All the collected records were checked by trained registered dietitians at the respective local center and then again at the study center.

A total of 1320 food and beverage items appeared in the DR. Intake of total energy and total fat were estimated based on the estimated intakes of all items and the STFCJ.^20^ *Trans* fatty acid intake was estimated based on the database created in the present study. Regarding intake of fast foods, baked goods, and confectionaries in the DR, fewer than 1% of subjects reported that these foods were home-cooked. We therefore considered these foods to be commercial foods, and calculated their intake using *trans* fatty acid values of the food products themselves (eg, french fries) rather than those of the raw food materials (potatoes and oil). For foods containing refined oil, margarine, and shortening eaten at restaurants (eg, pork cutlet), nutrient information was unavailable. We therefore calculated the *trans* fatty acid intake of these foods by summing the amount reported for the raw food materials (pork and lard).

### Statistical analyses

All statistical analyses were performed separately for women and men using SAS statistical software version 9.1 (SAS Institute Inc., Cary, NC, USA). We categorized subjects into 4 age groups (30–39 years, 40–49 years, 50–59 years, and 60–69 years). We also grouped subjects living in the 4 areas into 2 groups according to population density. Osaka (Osaka City: 11 743 persons/km^2^) and Okinawa (Ginowan City: 4446 persons/km^2^) had much higher population densities and were classified as urban; Nagano (Matsumoto City: 786 persons/km^2^) and Tottori (Kurayoshi City: 285 persons/km^2^) were classified as rural.^45^ Because no significant seasonal variation in *trans* fatty acid intake was observed in any analysis by age group or residential area (data not shown), all analyses were performed using the 16-day mean dietary intake of the subjects. Total fat intake was expressed as grams per day and percentage of total energy intake. *Trans* fatty acid intake was expressed as grams per day, percentage of total energy intake, and percentage of total fat intake. Differences between the urban and rural areas in total fat intake and *trans* fatty acid intake of subjects were examined using the non-paired *t*-test, while differences among age groups in total fat intake and *trans* fatty acid intake were determined using analysis of variance and the Tukey multiple comparison test. All reported *P* values are two-tailed, and a *P* value of <0.05 was considered statistically significant.

## RESULTS

Subject characteristics are shown in Table 2. Mean total energy intake was 1847 kcal/day for women and 2372 kcal/day for men. Mean total fat intake was 56.9 g/day (27.7% total energy) for women and 66.8 g/day (25.5% total energy) for men. Mean *trans* fatty acid intake was 1.7 g/day (0.8% total energy) for women and 1.7 g/day (0.7% total energy) for men.

**Table 2.** Characteristics of the 225 Japanese subjects

	Women (*n* = 119)			Men (*n* = 106)		
		
	Mean	SD	Range	Mean	SD	Range
Age (years)	49.7	11.1	30–69	50.4	10.8	30–69
Body height (cm)	154.6	6.2	132.5–170.7	168.0	6.4	150.0–186.0
Body weight (kg)	53.4	7.1	41.5–74.0	67.1	10.2	45.0–97.5
Body mass index (kg/m^2^)	22.3	2.8	17.8–31.3	23.7	2.9	17.4–30.9
Total energy intake (kcal/day)	1847	289	1143–3034	2372	389	1413–3473
Total fat intake (g/day)	56.9	11.3	33.2–101.1	66.8	12.4	40.7–100.9
Total fat intake (% total energy)	27.7	3.4	18.9–35.1	25.5	3.5	17.9–34.6
*Trans* fatty acid intake (g/day)	1.7	0.7	0.4–4.1	1.7	0.6	0.6–3.5
*Trans* fatty acid intake (% total energy)	0.8	0.3	0.3–1.9	0.7	0.2	0.2–1.2
*Trans* fatty acid intake (% fat)	2.9	0.9	1.4–6.5	2.5	0.7	1.1–4.3

Abbreviation: SD = standard deviation.

Major contributors to *trans* fatty acid intake were confectionaries, baked goods, and fats and oils. Approximately 75% of intake was attributable to industrially produced *trans* fatty acids (Table 3).

**Table 3.** Contribution (%) of selected food groups to total trans fatty acid intake among 225 Japanese subjects

Food Group^a^	Women (*n* = 119)		Men (*n* = 106)	
	
	Mean	SD	Mean	SD
Confectionaries (I)	21.7	19.7	15.3	25.0
Bakery (I)	19.1	13.3	18.0	13.7
Fats and oils (N)	2.0	2.0	2.1	2.4
Fats and oils (I)	14.1	6.0	20.0	7.4
Instant and retort foods (I)	7.5	6.4	10.0	8.3
Milk and dairy products (N)	12.3	8.2	9.7	7.9
Milk and dairy products (I)	3.3	4.1	3.1	5.1
Meat and meat products (N)	10.7	6.6	15.5	8.9
Margarine (I)	4.8	6.7	5.8	8.9
Fast foods (I)	3.5	7.0	3.7	8.8
Miscellaneous (I)	1.1	1.0	1.0	0.9

Abbreviation: SD = standard deviation; (I) = industrially derived; (N) = naturally derived.

^a^Food groups are defined in Table 1.

Mean total fat intake in urban subjects was significantly higher than that in rural subjects (women: 28.8% total energy vs 26.8% total energy, *P* = 0.001; men: 26.6% total energy vs 24.4% total energy, *P* = 0.001), as was mean *trans* fatty acid intake (women: 0.9% total energy vs 0.7% total energy, *P* = 0.004; men: 0.7% total energy vs 0.6% total energy, *P* = 0.004) (Table 4).

**Table 4.** Total fat and trans fatty acid intake of 225 Japanese subjects living in urban and rural areas^a^ of Japan

	Women (*n* = 119)					Men (*n* = 106)				
		
	Urban (*n* = 57)		Rural (*n* = 62)		*P* ^b^	Urban (*n* = 51)		Rural (*n* = 55)		*P* ^b^
				
	Mean	SD	Mean	SD		Mean	SD	Mean	SD	
Total energy (kcal/day)	1803	306	1888	268	0.11	2310	371	2436	400	0.11
Total fat (g/day)	57.7	11.8	56.2	10.8	0.47	68.0	12.0	65.8	12.7	0.36
Total fat (% total energy)	28.8	3.0	26.8	3.5	0.001	26.6	3.5	24.4	3.2	0.001
*Trans* fatty acid (g/day)	1.8	0.8	1.6	0.6	0.03	1.9	0.7	1.6	0.6	0.04
*Trans* fatty acid (% total energy)	0.9	0.3	0.7	0.3	0.004	0.7	0.2	0.6	0.2	0.004
*Trans* fatty acid (% fat)	3.1	1.0	2.7	0.8	0.02	2.7	0.8	2.4	0.6	0.049

Abbreviation: SD = standard deviation.

^a^According to population density,^45^ 4 residential areas were grouped into urban (Osaka and Okinawa) and rural (Nagano and Tottori) areas.

^b^Differences between subjects in the 2 areas were tested by the unpaired *t*-test.

Table 5 shows mean total fat and *trans* fatty acid intake by age group. Subjects aged 30–39 years had the highest mean total fat and *trans* fatty acid intake (women: 29.1% total energy and 1.0% total energy; men: 27.5% total energy and 0.8% total energy), followed by those aged 40–49 years, 50–59 years, and 60–69 years (*P* < 0.001). The Tukey *t*-test revealed that the mean total fat intake of women (% total energy) aged 30–39 years was significantly higher than that of women aged 60–69 years (*P* < 0.05). *Trans* fatty acid intake of both women and men aged 30–39 years was significantly higher than that of those aged 50–59 years and 60–69 years (*P* < 0.05), while the intake of women aged 40–49 years was significantly higher than that of those aged 60–69 years (*P* < 0.05).

**Table 5.** Total fat and trans fatty acid intake of 225 Japanese subjects according to age group

	Women (*n* = 119)									Men (*n* = 106)								
		
	30–39 years (*n* = 27)		40–49 years (*n* = 29)		50–59 years (*n* = 32)		60–69 years (*n* = 31)		*P* ^e^	30–39 years (*n* = 20)		40–49 years (*n* = 29)		50–59 years (*n* = 28)		60–69 years (*n* = 29)		*P* ^e^
								
	Mean	SD	Mean	SD	Mean	SD	Mean	SD		Mean	SD	Mean	SD	Mean	SD	Mean	SD	
Total energy																		
(kcal/day)	1879	384	1816	289	1849	215	1847	270	0.82	2241	303	2454	450	2454	382	2302	360	0.85
Total fat																		
(g/day)	60.6^a^	13.1	58.8^a,b^	12.4	55.0^a,b^	9.6	54.0^b^	9.3	0.01	68.5^a^	10.4	69.3^a^	14.1	69.4^a^	12.7	60.8^a^	9.5	0.02
(% total energy)	29.1^a^	2.9	29.0^a,b^	2.9	26.7^a,b^	3.1	26.7^b^	3.1	<0.001	27.5^a^	2.1	25.6^b,c,d^	4.1	25.6^b,c,d^	3.7	23.9^b,c,d^	2.7	<0.001
*Trans* fatty acid																		
(g/day)	2.1^a,b,c^	0.8	1.9^a,b,c^	0.8	1.7^a,b,c^	0.6	1.2^d^	0.4	<0.001	1.9^a,b,c^	0.5	1.9^a,b,c^	0.7	1.7^a,b,c^	0.6	1.4^d^	0.5	0.001
(% total energy)	1.0^a,b^	0.3	0.9^a,b,c^	0.3	0.8^b,c^	0.2	0.6^c,d^	0.2	<0.001	0.8^a,b^	0.2	0.7^a,b,c^	0.2	0.6^b,c^	0.2	0.6^c,d^	0.2	<0.001
(% fat)	3.4^a,b,c^	1.0	3.2^a,b,c^	0.8	3.0^a,b,c^	0.8	2.2^d^	0.6	<0.001	2.8^a,b,c^	0.6	2.7^a,b,c^	0.7	2.4^a,b,c^	0.6	2.4^d^	0.8	0.007

Abbreviation: SD = standard deviation.

^a,b,c,d^Values in the same row, but with different superscripts, are significantly different: *P* < 0.05 (Tukey multiple comparison test).

^e^Differences between age groups were tested by analysis of variance.

The distribution of *trans* fatty acid intake among 225 Japanese subjects is shown in Table 6. Twenty-four percent of women (*n* = 29) and 6% of men (*n* = 6) showed a mean intake of more than 1% of total energy intake. By area of residence, the frequency of *trans* fatty acid intake greater than 1% of total energy intake was higher among women living in urban areas (35%; *n* = 20) than in those living in rural areas (15%; *n* = 9). Among men, the frequency of *trans* fatty acid intake greater than 1% of total energy intake was higher in those living in urban areas (12%; *n* = 6) than in those living in rural areas (0%; *n* = 0). By age group, the frequency of *trans* fatty acid intake greater than 1% of total energy intake among women was higher in younger age groups: 30–39 years, 33% (*n* = 9); 40–49 years, 38% (*n* = 11); 50–59 years, 25% (*n* = 8); 60–69 years, 3% (*n* = 1). Among men, the frequency of *trans* fatty acid intake greater than 1% of total energy intake was similar among age groups: 30–39 years, 10% (*n* = 2); 40–49 years, 10% (*n* = 3); 50–59 years, 0% (*n* = 0); 60–69 years, 3% (*n* = 1).

**Table 6.** Distribution of trans fatty acid intake among 225 Japanese subjects^a^

	By living area				By age group								Total	
		
	Urban		Rural		30–39 years		40–49 years		50–59 years		60–69 years			
							
	*n*	%	*n*	%	*n*	%	*n*	%	*n*	%	*n*	%	*n*	%
Women	57	100	62	100	27	100	29	100	32	100	31	100	119	100
*Trans* fatty acid (g/day)														
0.47–0.99	4	7.0	11	17.7	0	0	1	3.4	2	6.3	12	38.7	15	12.6
1.00–1.49	16	28.1	24	38.7	6	22.2	10	34.5	10	31.3	14	45.2	40	33.6
1.50–1.99	18	31.6	16	25.8	11	40.7	6	20.7	13	40.6	4	12.9	34	28.6
2.00–2.49	7	12.3	5	8.1	2	7.4	4	13.8	6	18.8	0	0	12	10.1
2.50–2.99	4	7.0	5	8.1	4	14.8	4	13.8	0	0	1	3.2	9	7.6
3.00–4.08	7	12.3	2	3.2	4	14.8	4	13.8	1	3.1	0	0	9	7.6
*Trans* fatty acid (% total energy)														
0.31–0.49	3	5.3	11	17.7	0	0	0	0	3	9.4	11	35.5	14	11.8
0.50–0.74	16	28.1	27	43.5	6	22.2	12	41.4	10	31.3	15	48.4	43	36.1
0.75–0.99	18	31.6	15	24.2	12	44.4	6	20.7	11	34.4	4	12.9	33	27.7
1.00–1.24	11	19.3	4	6.5	5	18.5	4	13.8	6	18.8	0	0	15	12.6
1.25–1.49	6	10.5	4	6.5	2	7.4	5	17.2	2	6.3	1	3.2	10	8.4
1.50–1.95	3	5.3	1	1.6	2	7.4	2	6.9	0	0	0	0	4	3.4

Men	51	100	55	100	20	100	29	100	28	100	29	100	106	100
*Trans* fatty acid (g/day)														
0.68–0.99	6	11.8	5	9.1	0	0	0	0	5	17.9	6	20.7	11	10.4
1.00–1.49	13	25.5	21	38.2	4	20.0	11	37.9	7	25.0	12	41.4	34	32.1
1.50–1.99	12	23.5	19	34.5	7	35.0	9	31.0	9	32.1	6	20.7	31	29.2
2.00–2.49	9	17.6	6	10.9	6	30.0	4	13.8	3	10.7	2	6.9	15	14.2
2.50–2.99	7	13.7	2	3.6	2	10.0	1	3.4	3	10.7	3	10.3	9	8.5
3.00–3.49	4	7.8	2	3.6	1	5.0	4	13.8	1	3.6	0	0	6	5.7
*Trans* fatty acid (% total energy)														
0.20–0.49	10	19.6	17	30.9	0	0	8	27.6	7	25.0	12	41.4	27	25.5
0.50–0.74	20	39.2	27	49.1	9	45.0	9	31.0	16	57.1	13	44.8	47	44.3
0.75–0.99	15	29.4	11	20.0	9	45.0	9	31.0	5	17.9	3	10.3	26	24.5
1.00–1.23	6	11.8	0	0	2	10.0	3	10.3	0	0	1	3.4	6	5.7

^a^According to population density,^45^ 4 residential areas were grouped into urban (Osaka and Okinawa) and rural (Nagano and Tottori) areas.

Furthermore, we compared estimated *trans* fatty acid intake based on a database containing 1995 foods (1976 foods appearing in the STFCJ^20^ and 19 added foods) and that based on a database containing 1976 foods appearing in the STFCJ^20^ (without the addition of the 19 foods) to investigate the impact of these 19 foods. The mean *trans* fatty acid intake (g/day) calculated using the databases containing 1976 foods and 1995 foods was 1.5 g/day vs 1.7 g/day for women and 1.6 g/day vs 1.7 g/day for men. The mean *trans* fatty acid intake (% total energy) calculated using the databases containing 1976 foods and 1995 foods was 0.7% total energy vs 0.8% total energy for women and 0.6% total energy vs 0.7% total energy for men.

## DISCUSSION

We found that mean *trans* fatty acid intake was 0.8% of total energy among Japanese women and 0.7% of total energy among Japanese men. Twenty-four percent of women and 6% of men had a mean intake of more than 1% of total energy intake, the maximum recommended by the WHO^6^; the frequency was particularly high in women living in urban areas and in subjects aged 30–39 and 40–49 years.

The estimated mean *trans* fatty acid intake from 3 previous studies using oil production data or household data in Japan was 1.6^16^ and 1.8 g/capita/day^17^ (oil production data) and 0.7 g/day (household data).^18^ These studies did not include important foods containing *trans* fatty acids (ie, retort foods, fast foods, shortening, poultry, and traditional Japanese confectionaries). Three other studies using 24-hour recall or a diet record at a single point in time reported an estimated mean intake of 0.3 and 1.0 g/day,^15^^,^^18^^,^^19^ or 0.03% to 0.5% total energy^14^^,^^15^^,^^19^; however, in 1 study the selection and number of foods included in the database were not reported,^14^ in 2 studies the database was not developed specifically for Japanese populations and the number of foods included in the database was limited,^15^^,^^19^ and in 2 studies the sample size was small (*n* = 8; *n* = 25).^15^^,^^19^ One of these studies measured the 1-day diet (7-day diet records were initially collected and measurement was done for 1 of the 7 days) of 25 female students at a dietetic college, and yielded an intake estimate of 1.2 g/day (0.6% total energy).^19^ This result was lower than the present estimate, possibly due to differences in eating patterns between groups, given that important sources of *trans* fatty acids in the 7-day mean food group intake differed from those reported our subjects (pastry: 6.1 g vs 14.9 g in the present study; instant ramen noodles: 0.8 g vs 9.8 g; beef and organ meats: 9.9 g vs 17.0 g; milk and milk products: 184.0 g vs 145.7 g; margarine: 0.3 g vs 1.9 g; other fats and oils: 12.8 g vs 15.1 g; and confectionaries: 36.3 g vs 46.1 g). Further, the nutritional knowledge of subjects in the previous study may have influenced their eating habits.

The estimated mean *trans* fatty acid intake in the present subjects was within the range of estimated mean intake of residents of Western countries—1.2 to 7.1 g/day (0.5%–4.9% total energy)^1^^–^^5^^,^^46^^–^^52^—but relatively low. This difference in estimates may be due to the use of different databases with a limited numbers of foods, different dietary assessment methods, and/or different dietary habits. Regarding dietary sources of *trans* fatty acids, the contribution of industrially produced *trans* fatty acids in our subjects was approximately 75%, whereas that in Western countries ranged from 23% to 74%, although data on dietary sources were not available for all studies.^3^^–^^5^^,^^49^^–^^52^ This result indicates that the intake of industrially produced *trans* fatty acids (% total energy) in our subjects was comparable with that of most Western countries that have reported estimates,^1^^,^^2^^,^^46^^–^^48^ although the total *trans* fatty acid intake of our subjects was relatively low.

We acknowledge several limitations of our study. First, analytic *trans* fatty acid values were not available for all foods within a particular type, and variation in values occurred among food products within the same food group.^35^ In addition, although we added several foods which are important sources of *trans* fatty acids (ie, fast foods, baked goods, and confectionaries) to the database, these added foods do not represent all food products on the market. This limited the completeness of the database and, therefore, the assessment of *trans* fatty acid intake. Nevertheless, we were able to obtain all relevant data available from sources with suitably clear and comprehensive assessment methodologies, to carefully conduct matching processes for foods with similar food values, and to follow a similar process to that used in previous Western studies,^49^^,^^53^ which used standardized procedures^54^ to ensure database reliability. The mean *trans* fatty acid intake calculated with the database of 1976 foods (foods appearing in the STFCJ^20^) for women and men was 0.2 g/day (13%) or 0.1% total energy (14%) and 0.1 g/day (6%) or 0.1% total energy (17%), respectively; these values were lower than those calculated using the database of 1995 foods. In contrast, the use of the 2 databases to calculate % total energy yielded very similar values, indicating that the addition of a number of foods into the food composition database had a negligible effect when intake was expressed as % total energy. Second, although the use of diet records allows detailed assessment of the dietary intake of individuals, the self-reported dietary assessment method is subject to measurement error. Finally, our subjects were volunteers and, hence, not a representative sample of the general Japanese population. They may therefore have been more nutritionally conscious than others who did not participate in the study, and our results may thus not be generalizable to the entire Japanese population.

In conclusion, although mean *trans* fatty acid intake was below the maximum intake recommended by the WHO, the intakes of subgroups, especially women living in urban areas and people aged 30–39 and 40–49 years, was of concern. Further public health efforts to reduce *trans* fatty acid intake should be encouraged.
